# Clinical Characteristics and Viral RNA Detection in Children With Coronavirus Disease 2019 in the Republic of Korea

**DOI:** 10.1001/jamapediatrics.2020.3988

**Published:** 2020-08-28

**Authors:** Mi Seon Han, Eun Hwa Choi, Sung Hee Chang, Byoung-Lo Jin, Eun Joo Lee, Baek Nam Kim, Min Kyoung Kim, Kihyun Doo, Ju-Hee Seo, Yae-Jean Kim, Yeo Jin Kim, Ji Young Park, Sun Bok Suh, Hyunju Lee, Eun Young Cho, Dong Hyun Kim, Jong Min Kim, Hye Young Kim, Su Eun Park, Joon Kee Lee, Dae Sun Jo, Seung-Man Cho, Jae Hong Choi, Kyo Jin Jo, Young June Choe, Ki Hwan Kim, Jong-Hyun Kim

**Affiliations:** 1 Department of Pediatrics, Seoul Metropolitan Government-Seoul National University Boramae Medical Center, Seoul, Korea; 2 Department of Pediatrics, Seoul National University College of Medicine, Seoul, Korea; 3 Department of Pediatrics, Seoul Medical Center, Seoul, Korea; 4 Department of Pediatrics, Hongseong Medical Center, Hongseong, Korea; 5 Department of Pediatrics, Seongnam Citizens Medical Center, Seongnam, Korea; 6 Department of Pediatrics, Gyeonggi Provincial Medical Center Ansung Hospital, Ansung, Korea; 7 Department of Pediatrics, Seonam Hospital, Seoul, Korea; 8 Department of Pediatrics, Gyeonggi Provincial Medical Center Icheon Hospital, Icheon, Korea; 9 Department of Pediatrics, Dankook University Hospital, Cheonan, Korea; 10 Department of Pediatrics, Samsung Medical Center, Sungkyunkwan University School of Medicine, Seoul, Korea; 11 Department of Pediatrics, Masan Medical Center, Changwon, Korea; 12 Department of Pediatrics, Chung-Ang University Hospital, Seoul, Korea; 13 Department of Pediatrics, Busan Medical Center, Busan, Korea; 14 Department of Pediatrics, Seoul National University Bundang Hospital, Seongnam, Korea; 15 Department of Pediatrics, Chungnam National University Hospital, Daejeon, Korea; 16 Department of Pediatrics, Inha University Hospital, Incheon, Korea; 17 Department of Pediatrics, Myongji Hospital, Goyang, Korea; 18 Department of Pediatrics, Pusan National University Hospital, Busan, Korea; 19 Department of Pediatrics, Pusan National University Children’s Hospital, Yangsan, Korea; 20 Department of Pediatrics, Chungbuk National University Hospital, Cheongju, Korea; 21 Department of Pediatrics, Jeonbuk National University Medical School, Jeonju, Korea; 22 Department of Pediatrics, Dongguk University College of Medicine, Gyeongju, Korea; 23 Department of Pediatrics, Jeju National University Hospital, Jeju, Korea; 24 Department of Social and Preventive Medicine, Hallym University College of Medicine, Chuncheon, Korea; 25 Department of Pediatrics, Incheon St Mary's Hospital, The Catholic University of Korea, Incheon, Korea; 26 Department of Pediatrics, St Vincent's Hospital, The Catholic University of Korea, Suwon, Korea

## Abstract

**Importance:**

There is limited information describing the full spectrum of coronavirus disease 2019 (COVID-19) and the duration of severe acute respiratory syndrome coronavirus 2 (SARS-CoV-2) RNA detection in children.

**Objective:**

To analyze the full clinical course and the duration of SARS-CoV-2 RNA detectability in children confirmed with COVID-19 in the Republic of Korea, where rigorous public health interventions have been implemented.

**Design, Setting, and Participants:**

This case series of children with COVID-19 was conducted in 20 hospitals and 2 nonhospital isolation facilities across the country from February 18, 2020, to March 31, 2020. Children younger than 19 years who had COVID-19 were included.

**Exposures:**

Confirmed COVID-19, detected via SARS-CoV-2 RNA in a combined nasopharyngeal and oropharyngeal swab or sputum by real-time reverse transcription–polymerase chain reaction.

**Main Outcomes and Measures:**

Clinical manifestations during the observation period, including the time and duration of symptom occurrence. The duration of SARS-CoV-2 RNA detection was also analyzed.

**Results:**

A total of 91 children with COVID-19 were included (median [range] age, 11 [0-18] years; 53 boys [58%]). Twenty children (22%) were asymptomatic during the entire observation period. Among 71 symptomatic cases, 47 children (66%) had unrecognized symptoms before diagnosis, 18 (25%) developed symptoms after diagnosis, and only 6 (9%) were diagnosed at the time of symptom onset. Twenty-two children (24%) had lower respiratory tract infections. The mean (SD) duration of the presence of SARS-CoV-2 RNA in upper respiratory samples was 17.6 (6.7) days. Virus RNA was detected for a mean (SD) of 14.1 (7.7) days in asymptomatic individuals. There was no difference in the duration of virus RNA detection between children with upper respiratory tract infections and lower respiratory tract infections (mean [SD], 18.7 [5.8] days vs 19.9 [5.6] days; *P* = .54). Fourteen children (15%) were treated with lopinavir-ritonavir and/or hydroxychloroquine. All recovered, without any fatal cases.

**Conclusions and Relevance:**

In this case series study, inapparent infections in children may have been associated with silent COVID-19 transmission in the community. Heightened surveillance using laboratory screening will allow detection in children with unrecognized SARS-CoV-2 infection.

## Key Points

**Question:**

How long is severe acute respiratory syndrome coronavirus 2 (SARS-CoV-2) RNA detected in children, and are children with coronavirus disease 2019 (COVID-19) identifiable by symptoms?

**Findings:**

In this case series of 91 children with COVID-19 in Korea, 22.0% were asymptomatic. Only 8.5% of symptomatic cases were diagnosed at the time of symptom onset, while 66.2% had unrecognized symptoms before diagnosis and 25.4% developed symptoms after diagnosis; SARS-CoV-2 RNA was detected for a mean of 17.6 days overall and 14.1 days in asymptomatic cases.

**Meaning:**

Symptom screening fails to identify most COVID-19 cases in children, and SARS-CoV-2 RNA in children is detected for an unexpectedly long time.

## Teaser

This case series analyzes the full clinical course of coronavirus disease 2019 and duration of acute respiratory syndrome coronavirus 2 RNA detectability in children with confirmed infection in Korea.

## Introduction

Coronavirus disease 2019 (COVID-19) caused by severe acute respiratory syndrome coronavirus 2 (SARS-CoV-2) has spread to many countries, resulting in a global public health threat. The World Health Organization characterized COVID-19 as a pandemic on March 11, 2020, and as of April 20, 2 314 621 confirmed cases and 157 847 fatal cases have been reported.^1^

The Republic of Korea was one of the earliest countries struck by the COVID-19 outbreak, with the first imported case from China on January 20, 2020, and a sudden surge in COVID-19 cases associated with a religious group in the city of Daegu.^2,3^ As an effort to contain COVID-19 during this early outbreak with the largest case numbers after China, the Korea Centers for Disease Control and Prevention and local health departments used rigorous public health interventions, including prompt epidemiologic investigations, contact tracing followed by quarantine measures, and large-scale testing of all contacts, irrespective of symptom severity.^4^ Patients who were severely ill with COVID-19 were immediately hospitalized in designated hospitals. Patients with mild and asymptomatic cases were also put in isolation at city or provincial medical centers or community facilities called *living treatment centers*. This setting of strict quarantine and isolation of any contacts or confirmed cases provides a unique opportunity to understand the whole course of COVID-19 in children in detail.

As of April 20, 2020, a total of 10 674 cases have been confirmed, with 236 deaths in Korea.^5^ Among them were 718 children younger than 19 years (6.7% of total cases), and no child deaths have been reported to date since the first reported pediatric case.^6^ Recent reports on pediatric cases of COVID-19 suggest that children usually have mild illnesses.^7,8,9^ However, the full spectrum of illness in children is not clearly understood because many pediatric patients might not have been subject to viral testing. Furthermore, there is limited information regarding the duration of SARS-CoV-2 RNA detection in children’s respiratory tracts.^10^ This study aimed to analyze the clinical course and duration of SARS-CoV-2 RNA detection in children with COVID-19 in the setting of rigorous public health interventions.

## Methods

### Study Population

This study included children younger than 19 years who had confirmed COVID-19 in Korea from February 18 to March 31, 2020. Children were tested for COVID-19 when they had a history of close contact with confirmed cases, were epidemiologically linked to COVID-19 outbreaks, arrived from abroad, or had symptoms suspicious of COVID-19 as judged by physicians.^11^ All children with confirmed COVID-19 were placed in isolation at 20 hospitals and 2 nonhospital isolation facilities across the country. Cases linked to a specific religious group in Daegu were excluded, given limited clinical information. Cases of COVID-19 were diagnosed by detecting SARS-CoV-2 RNA in a combined nasopharyngeal and oropharyngeal swab or sputum by real-time reverse transcription–polymerase chain reaction (RT-PCR). The RT-PCR tests were performed at the Research Institute of Public Health and Environment of the city or provincial governments, delegate agencies, or hospitals, using COVID-19 test kits (Kogene Biotech, Seegene, and Biosewoom) approved by the Ministry of Food and Drug Safety. (Details of SARS-CoV-2 RT-PCR are provided in the eMethods in the Supplement.) The turnaround time of the tests was within 24 hours, and most were done within 6 to 12 hours. This study was approved by the institutional review board at each health care facility, except for 5 newly established isolation facilities, where an institutional review board is not operating. Informed consent was waived because the study involved no more than minimal risk to participants and could not be conducted without the waiver. The investigation was part of the public health response by the local health departments and was not subject to institutional review board approval.

### Data Collection

An electronic case report form was constructed, and physicians were asked to fill out the form. Physicians collected clinical data and viral test results of the patients and entered the data into each electronic case report form. Clinical data included age, sex, underlying disease, date of diagnosis, route of exposure, symptoms, laboratory test results, radiographic findings, treatment, and outcome. The route of exposure was based on the epidemiologic report by the local government using a standardized survey.

### Definitions

The date of confirmed diagnosis was defined as the date when an initial respiratory sample with a positive test result was collected. Health care isolation was lifted when patients had improvement in symptoms and 2 consecutive negative PCR results from nasopharyngeal and oropharyngeal swab specimens collected at least 24 hours apart. The date to discontinue isolation was defined as the date the second negative sample was collected. The duration of viral RNA detection was defined as the interval from the date of symptom onset to the date the first sample negative by PCR was collected. In asymptomatic individuals, the interval from the date of diagnosis was applied.

### Case Classifications

Patients were classified based on symptoms, signs, and findings of chest imaging. Children who did not develop any symptoms during the observation period were defined as asymptomatic. Children with fever (including mild fever) or respiratory symptoms with no infiltration on chest radiographs or chest computed tomography were defined as having upper respiratory tract infections (URTIs). Lower respiratory tract infection (LRTI) was defined by the presence of fever (including mild fever) or respiratory symptoms and any infiltrates on chest imaging. Patients were also categorized based on the severity of the disease. Mild cases were defined as the presence of any symptoms without abnormal chest imaging, while moderate cases were defined as the presence of LRTI. When a child with hypoxia had an oxygen saturation of less than 95%, required oxygen supplementation, or received mechanical ventilation, the patient was defined as having a severe case.

### Time and Duration of Symptom Occurrence

Patients who had any symptoms were categorized as those who had symptoms before diagnosis, those who developed symptoms at the time of diagnosis, and those who had been asymptomatic at diagnosis but later developed symptoms, based on the time between symptom onset and the time of diagnosis. The interval between the dates of symptom onset and diagnosis and the total duration of symptoms were determined for each group.

### Statistical Analysis

Percentages of clinical features were calculated from the number of patients with known information for that feature. The difference between normally distributed continuous variables was analyzed by the 2-sample *t* test, whereas nonnormally distributed variables were compared using the Mann-Whitney *U* test. Categorical variables were compared using the Pearson χ^2^ test or the Fisher exact test. The proportion of children in isolation over time by different symptomatic groups was compared by Kaplan-Meier survival analysis. The differences were statistically significant when the 2-sided *P* value was less than .05. The software program SPSS, version 26.0 (IBM) was used for statistical analysis.

## Results

### Epidemiology of COVID-19 in Children

**Figure 1.**
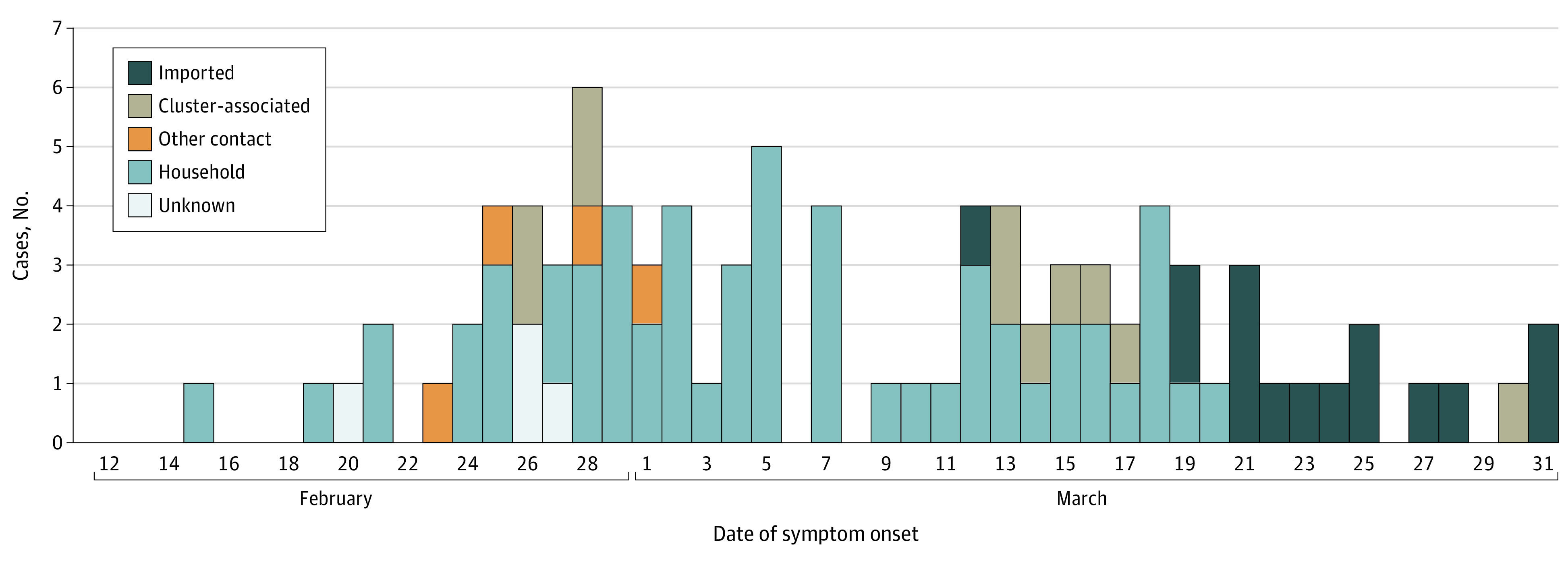
Epidemic Curve of Children With Coronavirus Disease 2019 in Korea From February 14 to March 31, 2020 Other contacts indicates close contact with a kindergarten teacher, care helper at a rehabilitation center, or with other individual with a confirmed case without a social relationship.

This study included 91 patients, accounting for 76.5% of all 119 children younger than 19 years in Korea whose cases were not connected to the outbreak of a specific religious group in Daegu, as reported by the Korea Centers for Disease Control and Prevention, and confirmed with COVID-19 from February 18 to March 31, 2020 (eFigure 1 in the Supplement). Nine patients’ information has been previously reported.^6,12,13^ The median age was 11 years (range, 27 days-18 years). Fifty-three patients (58%) were male, and 6 (7%) had underlying diseases (Table 1). No children had immunodeficiency. The most common source of infection was household contact (57 patients [63%]), followed by importation (15 [17%]), cluster-associated transmission (11 [12%]), other contacts (4 [4%]), and unknown sources (4 [4%]). Household contacts accounted for most cases from February 14 to March 18, 2020, while thereafter most cases were imported (Figure 1).

**Table 1.** Demographics of Children With Coronavirus Disease 2019

Characteristic	No. (%)^**a**^
Total No.	91
Sex	
Male	53 (58)
Female	38 (42)
Age, y	
Median (range)	11 (0.07-18)
<1	6 (7)
1-5	13 (14)
6-10	23 (25)
11-15	31 (34)
16-18	18 (20)
Underlying disease^b^	
None	85 (93)
Asthma	3 (3)
Epilepsy	3 (3)
Exposure route	
Household	57 (63)
Imported	15 (17)
Cluster-associated	11 (12)
Other contact^c^	4 (4)
Unknown	4 (4)

^a^ Percentages may not sum to 100% because of rounding.

^b^ No children had immunodeficiency.

^c^ Contacts with a kindergarten teacher or care helper at a rehabilitation center or close contacts with another individual with a confirmed case without a social relationship.

### Clinical Symptoms

Among 91 children with COVID-19, 20 (22%) remained asymptomatic throughout the monitoring period (Table 2). Patients who were symptomatic developed a wide range of symptoms. Fever (temperature, ≥38.0 °C) and mild fever (temperature, 37.5-<38.0 °C) developed in 27 patients (30%) and 35 patients (39%), respectively. Fifty-four children (60%) had respiratory symptoms, including cough, sputum, and rhinorrhea, whereas 16 (18%) presented with gastrointestinal symptoms, such as diarrhea and abdominal pain. There was 1 patient who only had abdominal pain and diarrhea, without fever or respiratory symptoms. Twelve children (16%) had loss of smell or taste. One patient only presented with loss of taste, without any other symptoms. Laboratory findings of the 91 patients were unremarkable (eTable in the Supplement).

**Table 2.** Clinical Manifestations of Children With Coronavirus Disease 2019

Clinical parameter	No./total No. (%)^a^
Symptoms	
None	20/91 (22)
Systemic	50/91 (55)
Fever (temperature, ≥38.0 °C)	27/91 (30)
Mild fever (temperature, 37.5-<38.0 °C)	35/91 (38)
Headache	12/77 (16)
Myalgia	7/77 (9)
Lethargy	5/89 (6)
Chills	3/90 (3)
Respiratory	54/90 (60)
Cough	37/90 (41)
Sputum	29/90 (32)
Rhinorrhea	24/90 (27)
Sore throat	22/77 (29)
Nasal stuffiness or congestion	8/90 (9)
Chest discomfort or pain	4/77 (5)
Dyspnea	1/77 (1)
Gastrointestinal	16/90 (18)
Diarrhea	11/90 (12)
Abdominal pain	6/77 (8)
Nausea or vomiting	6/90 (7)
Other	12/74 (16)
Loss of taste	8/74 (12)
Loss of smell	4/74 (5)
Eye pain	2/77 (3)
Disease severity^b^	
Asymptomatic	20/91 (22)
Mild	46/91 (51)
Moderate	20/91 (22)
Severe	2/91 (2)
Unclassified	3/91 (3)
Respiratory support	
Oxygen supplement	2/91 (2)
Mechanical ventilation	0/91
Intensive care management	0/91
Treatment^c^	
None	77/91 (85)
Lopinavir-ritonavir	13/91 (14)
Hydroxychloroquine	2/91 (2)
Mortality	0/91

^a^ Numbers may not sum to total because of missing data, and percentages may not sum to 100% because of rounding.

^b^ Mild cases were defined as the presence of any symptoms without abnormal chest imaging, moderate cases were defined as the presence of lower respiratory tract infection, and severe cases were defined as hypoxia with an oxygen saturation less than 95% or children with oxygen supplementation or mechanical ventilation. Cases are unclassified when chest imaging was not performed in patients with symptoms.

^c^ One patient received both lopinavir-ritonavir and hydroxychloroquine.

### Symptom Duration With Regard to the Date of Diagnosis

**Figure 2.**
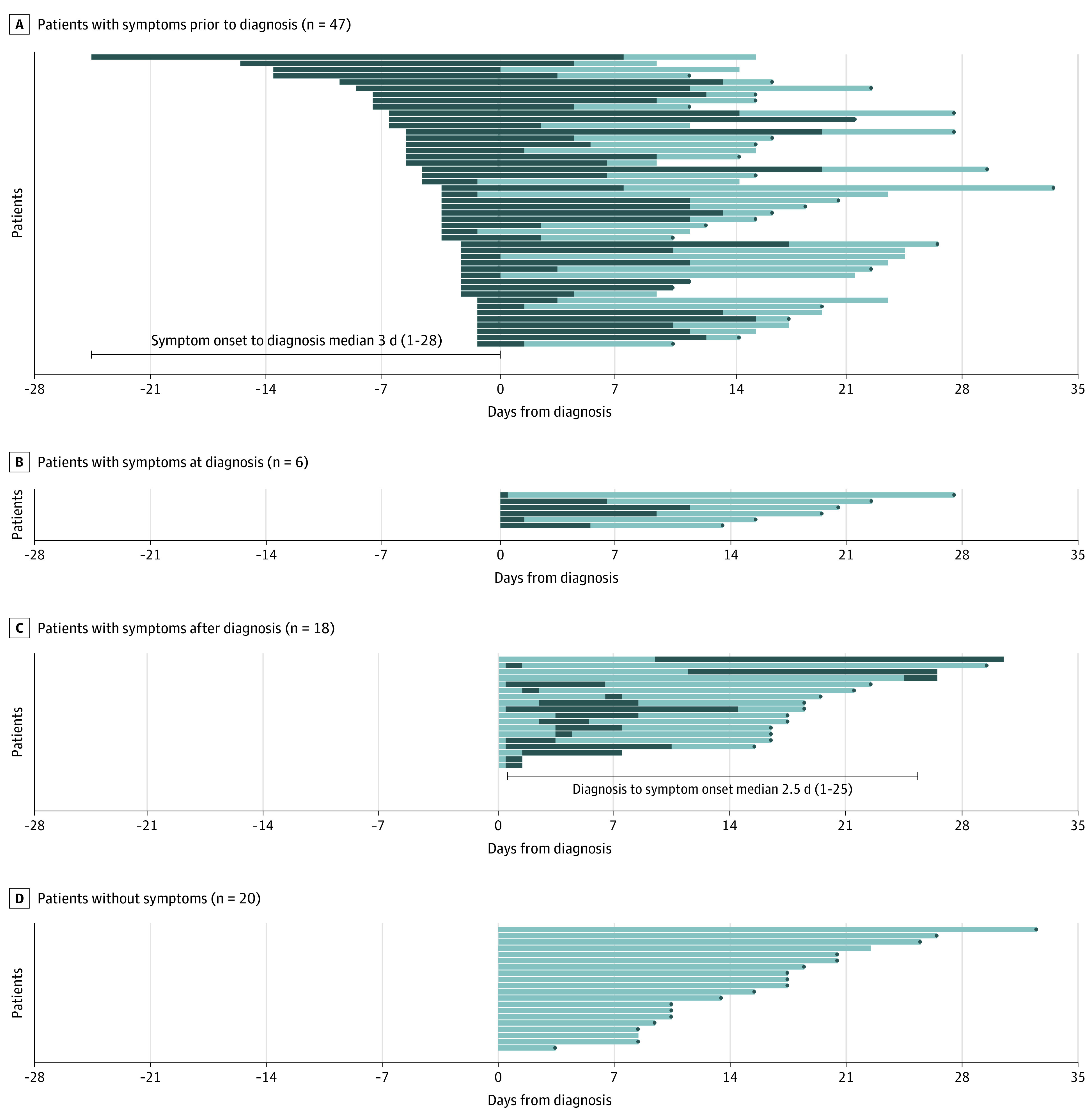
Time Course From Time of Diagnosis to End of Isolation in Children With Coronavirus Disease 2019 Time courses are presented according to the spectrum of symptom occurrence. Health care isolation was lifted when patients had improved symptoms and 2 consecutive negative polymerase chain reaction results from nasopharyngeal and oropharyngeal swab specimens collected at least 24 hours apart. The dark colors indicate the symptomatic period, and the light colors indicate the asymptomatic period. The gray dots indicate the date of release from isolation.

All 91 children with COVID-19 were monitored for a mean (SD) of 21.9 (8) days (Figure 2). Among them, 71 (78.0%) were symptomatic for a median (range) of 11 (1-36) days. The proportion of children with symptoms remaining at follow-up was 61% (43 of 71 children) at 7 days, 38% (27 of 71) at 14 days, and 10% (7 of 71) at 21 days. Forty-seven children (66%) had symptoms for a median (range) of 3 (1-28) days prior to diagnosis. The median (range) duration of symptoms was 13 (3-36) days. Six children (9%) started to have symptoms from the time of diagnosis that lasted for a median (range) of 6.5 (1-12) days. There were 18 patients (25%) who had been asymptomatic at the time of diagnosis and developed symptoms a median (range) of 2.5 (1-25) days after the diagnosis of COVID-19. These children had a median (range) symptom duration of 3.5 (1-21) days. Twenty (22%) children with COVID-19 remained asymptomatic during a monitored period with a mean (SD) of 16 (8) days.

### Duration of SARS-CoV-2 Detection in Children

**Figure 3.**
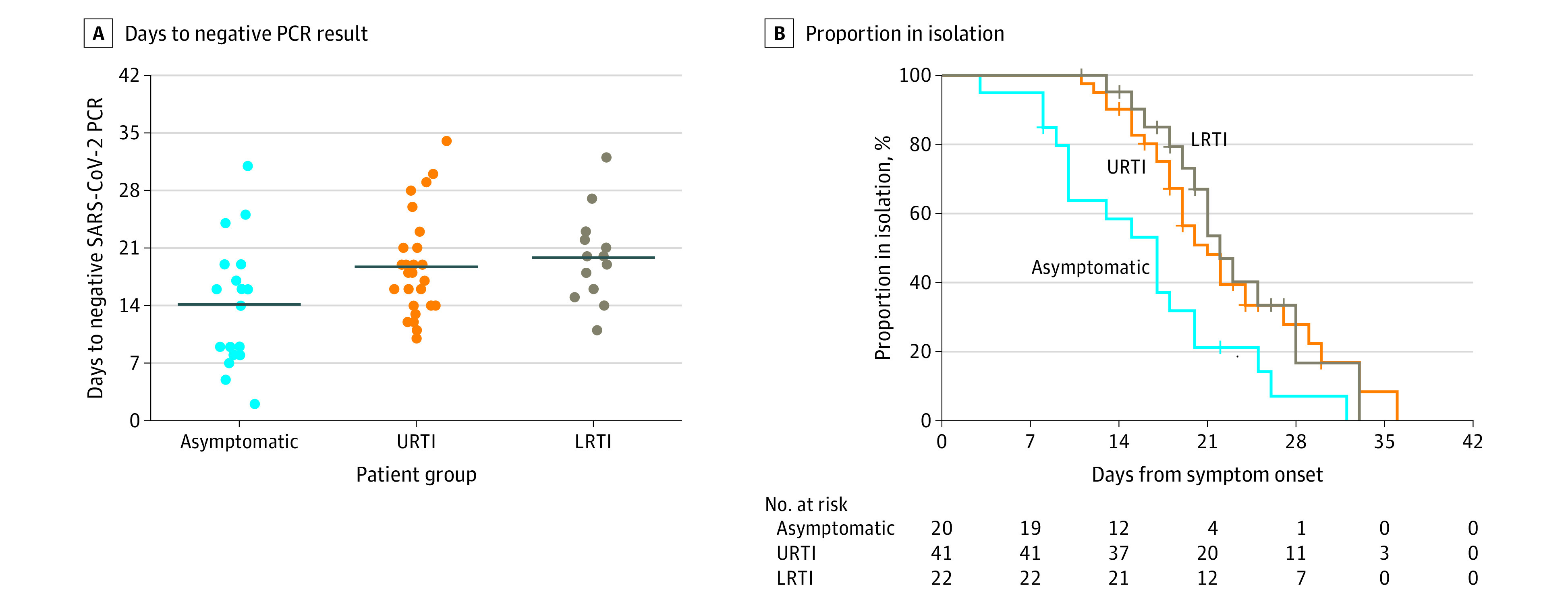
Duration From Symptom Onset to Negative Test Results and End of Isolation A, The mean duration from symptom onset to negative severe acute respiratory syndrome coronavirus 2 (SARS-CoV-2) polymerase chain reaction (PCR) results of the asymptomatic group and the groups with upper respiratory tract infection (URTIs) and lower respiratory tract infection (LRTIs). The line indicates the mean value. B, Kaplan-Meier curve for time to end of isolation. Health care isolation was lifted when patients had improved symptoms and 2 consecutive negative polymerase chain reaction results from nasopharyngeal and oropharyngeal swab specimens collected at least 24 hours apart. For asymptomatic cases, the number of days from diagnosis, instead of days from symptom onset, was applied.

Of 71 children with symptoms, 41 (58%) had URTIs, 22 (31%) had LRTIs, 3 (4.2%) did not undergo chest imaging because of mild illness, and the remaining 5 (7%) had other symptoms. The mean duration of virus RNA detection from symptom onset of the asymptomatic, URTI, and LRTI groups is shown in Figure 3A. Tests for SARS-CoV-2 were repeatedly performed in children at an interval with a median (range) of 3 (1-15) days to determine the cessation time of virus RNA detection. Overall, SARS-CoV-2 RNA was detected for a mean (SD) of 17.6 (6.7) days. The change of semiquantitative viral RNA load in 29 children with known RT-PCR cycle threshold values are shown in eFigure 2 in the Supplement. In asymptomatic cases, virus RNA was detected for a mean (SD) of 14.1 (7.7) days. Virus RNA in children with URTIs and LRTIs was detected for means (SDs) of 18.7 (5.8) days and 19.9 (5.6) days, respectively, and no significant difference was observed between the 2 groups (*P* = .54). Figure 3B shows the proportion of children placed in health care isolation over time in each group. After 14 days from symptom onset (or diagnosis in asymptomatic cases), 12 children (60%) with asymptomatic cases remained in isolation, and 37 patients (90%) and 21 patients (96%) with URTIs and LRTIs remained in isolation, respectively. By day 21 from symptom onset, 4 asymptomatic cases (20%) were in isolation, while 20 patients (49%) and 12 patients (55%) with URTIs and LRTIs remained in isolation, respectively.

### Severity of COVID-19

Apart from asymptomatic cases, 46 patients (65%) had mild cases and 20 (28%) had moderate cases (Table 2). There were 2 patients (3%) with severe cases of COVID-19, who were only supplemented with oxygen via nasal prong, without requiring mechanical ventilation.

### Treatment and Outcome

Twelve children (13%) with COVID-19 were treated with lopinavir-ritonavir, and 2 (2%) received hydroxychloroquine. Of those, 1 patient (1%) received both lopinavir-ritonavir and hydroxychloroquine (Table 2). The remaining 77 patients (85%) did not receive treatment for COVID-19. There was no difference in the mean (SD) duration of virus RNA detection between patients treated with lopinavir-ritonavir vs without lopinavir-ritonavir (17.9 [4.5] days vs 17.3 [8.3] days; *P* = .79). No fatal case was observed.

## Discussion

In the earlier epidemic of COVID-19 in China and the current outbreak in other countries, diagnostic tests were concentrated on patients who were hospitalized and those at high risk of complications, thus missing the opportunity to diagnose children with COVID-19 who were mildly ill or asymptomatic.^14^ However, the Korean health authorities and the local governments have responded to the COVID-19 pandemic by using massive testing of all contacts or suspected cases and strict individual-level case interventions (isolation, tracing, and quarantine).^4^ Children with confirmed COVID-19 were all put in isolation units regardless of the presence of symptoms or disease severity. This strategy enabled us to fully characterize the diverse spectrum of COVID-19 in children. In this study, most of the patients (79%), whose contact sources are known, had been monitored by the local health departments during their quarantine. The imported cases (17%) were evaluated through reinforced screening at quarantine checkpoints and SARS-CoV-2 testing at the airports.

The findings of this study have several important implications. Children with COVID-19 in this study presented with a wide range of symptoms and signs, which were not specific enough for COVID-19 to prompt diagnostic testing or anticipate disease severity. Recent studies on pediatric COVID-19 from China and the US demonstrated that most children present with mild symptoms or are asymptomatic, without experiencing severe clinical courses.^7,9,10^ Symptoms of URTI developed in 19% to 54% of the children whose cases were reported, while 16% to 28% were asymptomatic. A systematic review^15^ of 18 studies with 1065 children showed that children of any age were reported to have mild respiratory symptoms, namely fever, dry cough, and fatigue, or were asymptomatic. Comparable with these reports, approximately 60% of the children in this study had respiratory symptoms and 22% were asymptomatic. This study additionally described the presence of other symptoms, including gastrointestinal and sensory symptoms. Of note, 11% of children had loss of taste while 5% experienced anosmia. A recent study conducted in Europe reported that olfactory and gustatory dysfunctions were present in a much higher proportion of 86% and 88% of the adult COVID-19 patients, respectively.^16^ Whether these symptoms are highly associated with COVID-19 in children needs more research. In any case, symptoms in children in this study completely improved after a median of 11 days.

The major hurdle implicated in this study in diagnosing and treating children with COVID-19 is that a considerable number of children are asymptomatic, and even if symptoms are present, they are unrecognized and overlooked before COVID-19 is diagnosed. Approximately 52% of the children already had symptoms a median of 3 days prior to diagnosis. Given the very tight surveillance in Korea, it is notable that the symptoms of 70% of them, who had close contact with people with confirmed cases and were assumed to be in self-quarantine, were not recognized. Children in quarantine were tested for COVID-19 the day before they were released to confirm that they did not have the disease.^11^ Meanwhile, 42% of the children were asymptomatic at the time of diagnosis, and 47% of them were presymptomatic and later developed symptoms after a median of 2.5 days. The rest remained asymptomatic throughout the whole course. These data suggest that 93% of the children with COVID-19 could have been missed were it not for Korea’s intensive contact tracing and aggressive diagnostic testing. Cases of children with undocumented COVID-19 are worrisome because they could facilitate the rapid spread of SARS-CoV-2 in the community.^17,18^

Early detection is a key to contain the virus from spreading in the community. Unfortunately, the data from this analysis suggest that there is no other good alternative for early detection but to perform extensive virus testing. Considering that most children’s symptoms are too mild to be noticed, other countries fighting the pandemic would benefit from performing large-scale testing, followed by immediate isolation strategy when enacting quarantine. However, this strategy may be practical only when intense epidemiological investigation with contact tracing, high-quality tests with rapid turnaround time, and ample isolation facilities are available. Moreover, such heightened containment measures inevitably incur large expenses, as well as creating a shortage of health care workers and medical supplies.^19,20^ Psychiatric problems brought out by putting children with no symptoms in an isolation unit should also be considered.^21^ To solve this dilemma, national and international experts from various fields need to come together and work on effective countermeasures to combat the pandemic.

The uniqueness of this study is in its longitudinal analysis of virus RNA detection throughout the whole course of infection in children with COVID-19. No studies so far have thoroughly addressed on the duration of virus RNA detection and its difference by the presence of LRTIs in children, to our knowledge. In this study, SARS-CoV-2 RNA was detected for a mean of 17.6 days in children’s respiratory tracts. Children with URTIs and LRTIs had virus RNA detected for means of 18.7 days and 19.9 days, respectively, and the difference was not significant. It is alarming that the virus RNA can be detected even in children who are asymptomatic for a mean of 14.1 days. Studies in China and Singapore^22,23,24^ have reported transmission of SARS-CoV-2 by patients who are asymptomatic and even evidence of presymptomatic transmission in adults. Infectiousness of the virus could peak 2 to 3 days before symptom starts, and transmission during the presymptomatic stage is estimated to make up 44% of secondary infections.^25^ The detection of virus RNA in respiratory specimens in this study does not necessarily imply that viable virus is present. However, if proven infectious (because most of the children were asymptomatic, were presymptomatic, or had unrecognizable symptoms in this study), the transmission potential of SARS-CoV-2 in children and its effect on the community might be greater than expected.

### Limitations

The major limitation of this study is that we were not able to analyze the transmission potential of children with COVID-19. This is primarily because of Korea’s strict quarantine and isolation strategies, which minimize exposures to individuals with vulnerabilities. Thus, we could not answer the question about infectivity and the infectious period in this cohort study. In addition, we did not compare the duration of virus RNA detection between asymptomatic and symptomatic groups, because determining the start of RNA detection in children who were asymptomatic was not possible. Moreover, follow-up tests for SARS-CoV-2 RNA were not performed at uniform intervals in all patients; hence, the duration of SARS-CoV-2 RNA detection might not be exact. Although we did not include children connected to the outbreak of a specific religious group, the result of our study is considered to represent the entire picture of children with COVID-19 in Korea.

## Conclusions

In conclusion, the findings of this study suggest that suspecting and diagnosing COVID-19 in children based on their symptoms without epidemiologic information and virus testing is very challenging. Most of the children with COVID-19 have silent disease, but SARS-CoV-2 RNA can still be detected in the respiratory tract for a prolonged period. The potential role of children in transmitting disease in the community needs to be further elucidated, and strategies to contain COVID-19 should reflect its effects. Heightened surveillance using laboratory screening will allow detection in children with unrecognized SARS-CoV-2 infection.

## Notes

Supplement. **eMethods.** Detection of Severe Acute Respiratory Syndrome Coronavirus 2 (SARS-CoV-2) **eFigure 1.** Flow diagram for children with COVID-19 included in this study **eFigure 2.** Changes in viral RNA load in nasopharyngeal swabs of children with COVID-19 **eTable.** Laboratory Findings of Children with COVID-19
