# Supplementary material for: Clinical Characteristics and Viral RNA Detection in Children With Coronavirus Disease 2019 in the Republic of Korea
Source: JAMA Pediatr. 2020 Aug 28;175(1):73–80. doi: 10.1001/jamapediatrics.2020.3988 (PMC7455883; doi:10.1001/jamapediatrics.2020.3988)
Supplement: Supplement. — eMethods. Detection of Severe Acute Respiratory Syndrome Coronavirus 2 (SARS-CoV-2) eFigure 1. Flow diagram for children with COVID-19 included in this study eFigure 2. Changes in viral RNA load in nasopharyngeal swabs of children with COVID-19 eTable. Laboratory Findings of Children with COVID-19 [file jamapediatr-e203988-s001.pdf]

Supplementary Online Content

Han MS, Choi EH, Chang SH, et al. Clinical characteristics and viral RNA detection in children with coronavirus disease 2019 in the Republic of Korea. *JAMA Pediatr*. Published online August 28, 2020. doi:10.1001/jamapediatrics.2020.3988

eMethods. Detection of Severe Acute Respiratory Syndrome Coronavirus 2 (SARS-CoV-2)

eFigure 1. Flow diagram for children with COVID-19 included in this study

eFigure 2. Changes in viral load in nasopharyngeal swabs of 29 children with COVID-19

eTable. Laboratory Findings of Children with COVID-19

This supplementary material has been provided by the authors to give readers additional information about their work.

©2020 Han MS et al. *JAMA Pediatrics.*

eMethods. Detection of Severe Acute Respiratory Syndrome Coronavirus 2 (SARS-CoV-2)

RNA was extracted from the respiratory samples of patients with coronavirus disease (COVID-19) and real-time reverse transcription polymerase chain reaction was performed in the Research Institute of Public Health and Environment of the city/provincial governments, delegated agencies, or hospitals using COVID-19 test kits. The assay used in the Research Institute of Public Health and Environment detects two genes, the E gene and the RNA-dependent RNA polymerase (RdRp) region of the ORF1b gene. The E gene is used for the screening test, and the RdRp gene is used for the confirmatory test. COVID-19 test kits from three producers were used in the delegated agencies and hospitals in this study and the product name and the target genes are as follows.

Producer	Product	Target genes
Kogene Biotech	PowerChek 2019-nCoV Real-time PCR Kit	E, RdRp
Seegene	AllPlex 2019-nCoV Assay	E, RdRp, N
Biosewoom	Real-Q 2019-nCoV Detection kit	E, RdRp

Primer and probe used to detect SARS-CoV-2 are as follows. Data are provided by the Korean Centers for Disease Control and Prevention and the World Health Organization.

Primer & Probe	Sequence (5'→3')	Target gene
E_Sarbeco_F1	ACAGGTACGTTAATAGTTAATAGCGT	E
E_Sarbeco_R2	ATATTGCAGCAGTACGCACACA	
E_Sarbeco_P1	FAM–ACACTAGCCATCCTTACTGCGCTTCG–BHQ	
RdRP_SARSr-F2	GTGARATGGTCATGTGTGGCGG	RdRp
RdRP_SARSr-R1	CARATGTTAAASACACTATTAGCATA	
RdRP_SARSr-P2	FAM–CAGGTGGAACCTCATCAGGAGATGC–BHQ	
N_Sarbeco_F1	CACATTGGCACCCGCAATC	N
N_Sarbeco_R1	GAGGAACGAGAAGAGGCTTG	
N_Sarbeco_P1	FAM–ACTTCCTCAAGGAACAACATTGCCA–BBQ	

eFigure 1. Flow diagram for children with COVID-19 included in this study

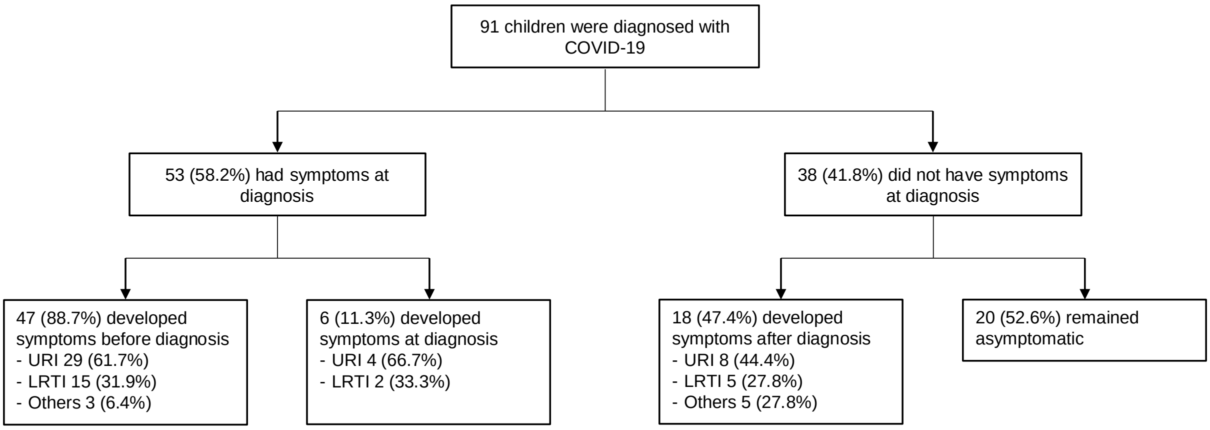

COVID-19, coronavirus disease; URTI, upper respiratory tract infection; LRTI, lower respiratory tract infection

©2020 Han MS et al. *JAMA Pediatrics.*

eFigure 2. Changes in viral load in nasopharyngeal swabs of 29 children with COVID-19

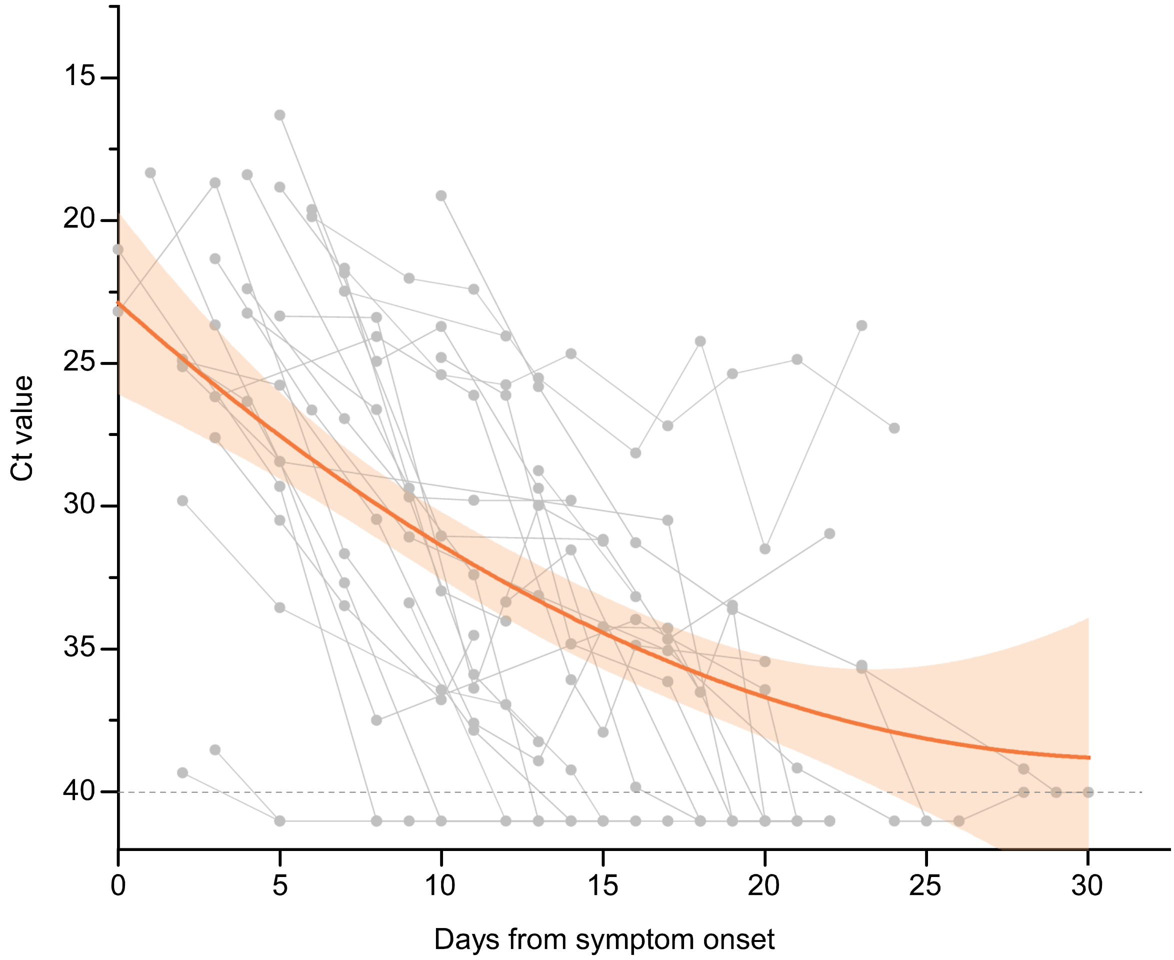

Cycle threshold (Ct) values of RdRp on real-time reverse transcription polymerase chain reaction are shown in gray. The limit of detection is a Ct value of 40, and specimens with undetectable values are presented under the dashed line. The thick red line indicates trend in viral load over time, and the shaded areas represent 95% confidence intervals.

©2020 Han MS et al. *JAMA Pediatrics.*

eTable. Laboratory Findings of Children with COVID-19

	Total (N = 91)	URTI (n = 44)	LRTI (n = 22)	*P*
WBC (/μL), mean (±SD)	6,131±2,322	6,583±2,815	5,819±1,893	0.074
ANC (/μL), mean (±SD)	2,827±1,461	2,808±1,458	2,949±1,589	0.569
ALC (/μL), mean (±SD)	2,514±1,446	2,847±1,782	2,134±866	0.005
aPTT (sec), mean (±SD)	31.3±7.9	29.5±6.5	35.0±6.2	0.043
PT (sec), mean (±SD)	14.1±5.7	13.3±4.6	12.8±0.9	0.649
PT (INR), mean (±SD)	1.06±0.06	1.05±0.05	1.06±0.07	0.874
T.bil (mg/dL), mean (±SD)	0.8±1.4	1.2±2.1	0.6±0.3	0.979
AST (IU/L), mean (±SD)	27±12	29±12	26±13	0.111
ALT (IU/L), mean (±SD)	23±19	22±13	27±26	0.642
BUN (mg/dL), mean (±SD)	11±3	11±3	11±3	0.674
Cr (mg/dL), mean (±SD)	0.59±0.20	0.56±0.21	0.62±0.18	0.128
CRP (mg/dL), mean (±SD)	0.25±0.61	0.19±0.29	0.41±0.90	0.341
LDH (IU/L), mean (±SD)	256±103	263±89	261±121	0.110

URTI, upper respiratory tract infection; LRTI, lower respiratory tract infection; WBC, white blood cell count; ANC, absolute neutrophil count; ALC, absolute lymphocyte count; aPTT, activated partial thromboplastin time; PT, prothrombin time; T.bil, total bilirubin; AST, aspartate transaminase; ALT, alanine transaminase; BUN, blood urea nitrogen; Cr, creatinine; CRP, C-reactive protein; LDH, lactate dehydrogenase.

©2020 Han MS et al. *JAMA Pediatrics.*
